# Climate for evidence-based mental health care implementation in Germany: psychometric investigation of the Implementation Climate Scale (ICS)

**DOI:** 10.1038/s41598-023-32282-4

**Published:** 2023-03-31

**Authors:** Katharina Szota, Hanna Christiansen, Gregory A. Aarons, Mark G. Ehrhart, Anne Fischer, Rita Rosner, Regina Steil, Antonia Barke

**Affiliations:** 1 Department of Psychology, Philipps-University of Marburg, Gutenbergstr. 18, 35032 Marburg, Germany; 2 Department of Psychiatry, University of California, 9500 Gilman Drive (0812), La Jolla, San Diego, CA 92093-0812 USA; 3 Department of Psychology, University of Central Florida, P.O. Box 161390, Orlando, FL 32816-1390 USA; 4 Department of Psychology, Goethe University Frankfurt, Varrentrappstr. 40-42, 60486 Frankfurt am Main, Germany; 5 Department of Clinical and Biological Psychology, Catholic University of Eichstätt-Ingolstadt, Levelingstr. 7, 85049 Ingolstadt, Germany; 6 Division of Clinical Psychology and Psychological Intervention, Institute of Psychology, University of Duisburg-Essen, Universitätsstr. 2, S06 S03 B24, 45141 Essen, Germany

**Keywords:** Health policy, Health services, Psychology

## Abstract

Organizational implementation climate is an important construct in implementation research to describe to what extent implementation is expected, supported, and rewarded. Efforts in bridging the research-practice gap by implementing evidence-based practice (EBP) can benefit from consideration of implementation climate. The Implementation Climate Scale (ICS) is a psychometrically strong measure assessing employees’ perceptions of the implementation climate. The present cross-sectional study aimed at providing a German translation and investigating its psychometric properties. The translation followed standard procedures for adapting psychometric instruments. German psychotherapists (*N* = 425) recruited online completed the ICS, the Evidence Based Practice Attitudes Scale (EBPAS-36D) and the Intention Scale for Providers (ISP). We conducted standard item and reliability analyses. Factorial validity was assessed by comparing an independent cluster model of Confirmatory Factorial Analysis (ICM-CFA), a Bifactor CFA, a Second-order CFA and an (Bifactor) Exploratory Structural Equation Model (ESEM). Measurement invariance was tested using multiple-group CFA and ESEM, convergent validity with correlation analysis between the ICS and the ISP subjective norms subscale (ISP-D-SN). The mean item difficulty was *p*_i _= .47, mean inter-item correlation *r* = .34, and mean item-total correlation *r*_itc_ = .55. The total scale (ω = 0.91) and the subscales (ω = .79–.92) showed acceptable to high internal consistencies. The model fit indices were comparable and acceptable (Second-order CFA: RMSEA [90% CI] = .077 [.069; .085], SRMR = .078, CFI = .93). Multiple-group CFA and ESEM indicated scalar measurement invariance across gender and presence of a psychotherapy license. Psychotherapists in training reported higher educational support for EBP than licensed psychotherapists (*T* = 2.09, *p* = .037, *d* = 0.25). The expected high correlation between the ICS and the ISP-D-SN was found (*r* = .59, *p* < .001). Results for the German ICS confirm good psychometric properties including validity.

## Introduction

Evidence-based practice (EBP) aims to integrate “the best research evidence with clinical expertise and patient values”^1^ when providing health and medical services. EBP implementation in routine care has shown to improve outcomes for patients^2^ as well as cost-effectiveness of interventions for health care systems^3–5^. Still, there is evidence for a persistent research-practice gap^6,7^. This has given rise to a global focus on research efforts in order to investigate barriers and facilitators of the dissemination and implementation of EBP in health care^8^. Adequate measurement of implementation mechanisms is required to advance this research area. It should have a conceptual, theoretical, and empirical basis, show good psychometric qualities, and be pragmatic^9^.

Implementation theories and frameworks have been developed to provide consistent definitions of relevant constructs and a basis for empirical investigations of the relationships between these constructs^10^. These frameworks are valuable for guiding the planning, design and process of hands-on efforts to implement EBP^11^. One widely used implementation framework is the Exploration, Preparation, Implementation, Sustainment (EPIS) framework^11,12^: The framework describes these four phases of the implementation process and identifies especially relevant factors during these phases: the outer system, inner context, bridging factors and innovation factors. Within the inner context, both organizational characteristics (e.g., the organizational implementation climate) and individual adopter characteristics (e.g., providers’ attitudes towards EBP implementation) are highlighted as central to the implementation success during the exploration and active implementation phase^12^.

Implementation climate is defined as “employees’ shared perceptions of the importance of innovation implementation within the organization”^13^, or whether “the adoption, implementation, and use of an innovation such as EBP is expected, rewarded, and supported by the organization”^14^. This organizational factor consistently appears to improve the implementation success^15–18^. A 5-year panel analysis indicated that improvements of the implementation climate resulted in increased EBP use by clinicians^18^. Although implementation research is often criticized for relying on self-report measures when assessing implementation outcome, two recent studies that use expert and observer ratings support the assumption that implementation climate predicts the adherence to EBP^19,20^. The implementation climate, in turn, is associated and thought to be influenced by other organizational factors, for example leadership^16,21–23^. Moreover, a stronger implementation climate is associated with more positive attitudes towards the adoption of EBP among the providers working within the organization ^22,24^.

One pragmatic instrument designed to assess the implementation climate of organizations or units within organizations such as a clinic or team is the Implementation Climate Scale (ICS) ^14^. It was developed based on review of existing literature and through a participatory process in collaboration with subject matter experts (e.g., experts in leadership and climate, clinical program managers, and clinicians). Building upon this past research, it is assumed that organizations that are willing to create an optimal organizational climate for EBP implementation (1) emphasize the priority of EBP implementation, (2) provide educational support for the EBPs, (3) recognize and (4) provide rewards for employees that use EBPs, and select employees that are experienced with the use of EBPs (5) or are willing to adopt new practices (6)^14^. The 18 items of the ICS comprise six subscales that are then aggregated to represent the overall implementation climate (see Table 1). Accordingly, the factor structure could best be regarded as a Second-order model. The items are designed to be group-referenced, that is referring to the unit level of interest. For example, questions may be phrased to refer to the whole organization, to a specific clinic, or some other organizational unit (e.g., hospital, health center, etc.). The ICS was designed originally in the mental health care context and has subsequently shown strong psychometric properties in a wide range of contexts from substance use disorder treatment organizations^25^, child welfare^26^, nursing^27^ and education settings^28^. A Norwegian study indicated cross-cultural validity in a translated version^29^.

**Table 1:** ICS Subscales with item examples.

Subscale	Item example
(1) Focus on EBP	“Using evidence-based practices is a top priority in this team/agency”
(2) Educational support for EBP	“This team/agency provides evidence-based practice training materials, journals, etc.”
(3) Recognition for EBP	“Clinicians who use evidence-based practices are held in high esteem in this team/agency”
(4) Rewards for EBP	“This team/agency provides financial incentives for the use of evidence-based practices”
(5) Selection for EBP	“This team/agency selects staff who have previously used evidence-based practices”
(6) Selection for openness	“This team/agency selects staff open to new types of interventions”

*ICS* Implementation Climate Scale, *EBP* Evidence-based Practice.

So far, no German translation of the ICS exists. This is unfortunate in view of the rising importance and efforts of implementation research in German-speaking countries and the associated need for reliable and valid German instruments assessing implementation constructs^30^. Regarding evidence-based mental health care for example, implementation research is growing in relevance and attention in German-speaking countries^31,32^. In Germany, a research opportunity for the implementation of EBP arises in the context of the regulation of psychotherapy practice by law^33^. The relevant legislation (‘Psychotherapeutengesetz’, PTG)^33^ was enacted on 1 January 1999. It provides legal protection of the title ‘psychotherapist’ and regulates the training requirements. In addition, it stipulates that psychotherapy must be based on scientific evidence (decided about by a Scientific Advisory Board of the Federal Medical Chamber and the Federal Psychotherapeutic Chamber)^33^. The training requirements to become a licensed psychotherapist include a medical, psychological, or educational degree followed by a period of practical postgraduate training^33,34^. Once psychotherapists are licensed, they can provide outpatient services working in private practices with reimbursement by the statutory (or private) health insurance. Besides outpatient services, a substantial part of the mental health care in Germany is offered as inpatient services by specific hospitals^33,35^. An amendment of the PTG was approved in September 2019, aiming to further align the training for psychotherapy to the existing structure of medical education^34^. Psychotherapy training always had to take place at state approved training sites. In the future, pre-graduate training will be restricted to universities. Whether this will be helpful to close the existing research-practice gap and will lead to an accelerated uptake of EBP in mental health care will be an important field of research during the next years. The assessment of implementation climate may help to inform understanding of determinants and mechanisms of more or less successful strategies and arrangements in this context^36^.

The goal of the present study was to translate the ICS into German and examine its psychometric properties. Based on survey data from a sample of German psychotherapists, the translated ICS was evaluated on the individual practitioner level in terms of item analysis, scale and subscale reliability, comparison of different factor models, assessment of measurement invariance across gender groups and psychotherapy license status. Convergent validity was assessed based on correlations of the ICS with the individual behavioral intentions for EBP use, with the expectation that behavioral intentions, and particularly ratings of subjective norms, would be higher when climate perceptions were higher. Further analyses included examining the relationships between the ICS and attitudes towards EBP, as well as several demographic characteristics (age, gender, license status, and in-patient vs. outpatient setting).

## Methods

### Procedure

#### Translation

The ICS was translated in accordance with the WHO recommendations (www.who.int/substance_abuse/research_tools/translation/en/). The ICS was translated into German by the first author (KS) and back-translated by the bilingual English-speaking senior author (AB). The original authors (MGE, GAA) of the scale reviewed back translated items to ensure that they reflected the original constructs. Item 11 was adjusted to fit the German mental healthcare system, which does not offer any wage increases when EBP is implemented. The original and back-translated versions were then reviewed in a consensus meeting of the translating authors. A group of German clinical psychotherapists (in training) and researchers (*n* = 26) as well as a graduate linguist reviewed this consented version for comprehensibility and wording. Their suggestions were discussed and considered by the translating authors in a second consensus meeting, resulting in a final German version of the scale (see Supplemental material 1).

#### Recruitment and data collection

All data were collected online via an openly accessible online survey, using the scientific survey platform SoSci Survey (www.soscisurvey.de). The survey was online and accessible from 14th November 2019 to 27th April 2020. The link to the online survey was widely distributed via e-mail lists of professional psychotherapy organizations that all licensed psychotherapists are members of, universities, training institutes, and psychiatric in- and outpatient institutions as well as Facebook groups of psychotherapists. On the last page of the survey, participants could opt to receive information about the study results or take part in a raffle. If they chose one or both of these options, they were linked to a separate page where they could register their e-mail addresses independently of their survey answers.

### Ethics

The Internal Review Board of the University of Marburg approved the online survey study (approval number: 2019-58 k). All methods were performed in accordance with the institutional guidelines. Participants provided informed consent after receiving study information and before they were able to access the survey. All raw data were stored securely at the Department of Psychology at Philipps University in Marburg, Germany, and were collected anonymously.

### Participants

Eligible participants were licensed psychotherapists for adults, children, and adolescents as well as psychotherapists enrolled in postgraduate training to obtain such a license. Therefore, all participants had medical, psychological, or educational degrees. No exclusion criteria except other profession were applied. The link to the online survey was visited 2,417 times. Overall, 913 participants continued after providing informed consent. Of these, 863 met the inclusion criteria (i.e., profession). A total of 251 participants were excluded because they discontinued the survey before completing the ICS, five due to conspicuous response patterns in the ICS (e.g., straight-lining despite reverse coded items), two due to implausible answers (e.g., being 99 years old). A total of 180 licensed psychotherapists (29.8% of the total sample) stated working in private practices. These participants (80.6% female, *M* = 48.1 years, *SD* = 11.9) were excluded from further analyses since the ICS captures the implementation climate of organizations and work group.

The final analysis sample consisted of 425 participants that stated working in organizations and work groups and thus completed the ICS, with an age of 23 to 61 years (*M* = 31.9, *SD* = 6.6) and consisting of 362 (85.2%) women and 62 (14.6%) men. A large portion of the sample reported working in science (44.2%) or stated being in postgraduate training to become psychotherapists (79.4%); 97.9% reported a German nationality. For further information regarding the samples’ professional characteristics, see Table 2.

**Table 2:** Demographics and information on profession for the sample.

Therapy orientation	*N* (%)	Professional group	*N* (%)	Current occupation	*N* (%)
Cognitive behavior therapy (CBT)	338 (76.5)	Psychotherapist in training	245 (57.6)	Outpatient practice	215 (50.8)
Psychodynamic psychotherapy (PDT)	58 (13.7	Licensed psychotherapist	53 (12.5)	Psychiatric hospital	85 (20.1)
PDT and psychoanalysis	13 (3.1)	Child and adolescent psychotherapist	34 (8.0)	Psychiatric day-clinic	47 (11.1)
CBT and systemic therapy	9 (2.1)	Child and adolescent psychotherapist in training	92 (21.6)	Clinic for psychosomatic medicine	36 (8.5)
Other	4 (0.9)	Other	1 (0.2)	Rehabilitation clinic/center	26 (6.1)
				University	12 (2.8)
				Other	23 (5.4)

*CBT* Cognitive behavior therapy, *PDT* Psychodynamic psychotherapy.

### Measures

#### Demographics and information on training and profession

Participants provided information on age, gender and nationality as well as their education and occupation. In detail, we asked participants about their university degree, license status, therapy orientation, and current occupation and work context.

#### Implementation Climate Scale (ICS)

The ICS is an 18-item instrument measuring the implementation climate in organizations and work groups^14^. Respondents were asked to rate their agreement with statements describing the climate with regard to the implementation of EBP in the organization or work group that they currently work for. A 5-point Likert scale ranging from 0 (‘not at all’) to 4 (‘to a very great extent’) is used. Six subscales can be calculated: Focus on EBP (α = 0.91 in our sample); Educational Support for EBP (α = 0.85); Recognition for EBP (α = 0.80); Rewards for EBP (α = 0.78); Selection for EBP (α = 0.84); Selection for Openness (α = 0.78). The total scale (α = 0.90) is created by computing means of the subscales. Higher scores (range 0 to 72 for the total scale) indicate a more favorable rating of the organizations’ implementation climate. The German instrument including scoring instructions can be found in the Supplemental material 1.

#### Evidence-based practice attitudes scale (EBPAS-36D)

The EBPAS-36 was developed to assess mental health and social service providers’ attitudes towards adopting EBP^37^ and a validated German translation is available^38^. The 36 items form 12 subscales of three items each: Requirements (α = 0.89 in our sample), Appeal (α = 0.69), Openness (α = 0.75), Divergence (α = 0.65), Limitations (α = 0.82), Fit (α = 0.68), Monitoring (α = 0.77), Balance (α = 0.65), Burden (α = 0.81), Job security (α = 0.89), Organizational support (α = 0.85), and Feedback (α = 0.76). Respondents are asked to rate their agreement with statements on a 5-point Likert scale ranging from 0 (‘not at all’) to 4 (‘to a very great extent’). Most items are worded in a way that higher scores indicate more positive attitudes towards the adoption of EBP; 15 items are scored reversely. A mean of the subscales can be computed to create a total scale (α = 0.89).

#### Intention scale for providers-direct items (ISP-D)

The ISP is a 70-item instrument assessing individual behavioral intentions for EBP use^39^ based on the theory of planned behavior^40^. The Direct Items measure of the ISP was previously investigated regarding its psychometric properties^41^. It consists of 16 items that assess attitudes (ISP-D-A, 5 items, α = 0.74 in our sample, higher scores indicating more negative attitudes), subjective norms (ISP-D-SN, 3 items, α = 0.84, higher scores indicating greater perceived social pressure to perform EBP), perceived behavioral control (ISP-D-PBC, 4 items, α = 0.71 in our sample, higher scores indicating a higher perceived control to perform EBP) and behavioral intention to use EBP (ISP-D-BI, 4 items, α = 0.88 in our sample, higher scores indicating a higher readiness to implement EBP). Responses are given on 7-point rating scales. The original English version was translated into German by the first author (KS) and back-translated by the bilingual English-speaking senior author (AB).

### Statistical analysis

The statistical analyses were performed using IBM SPSS 28 for Windows (Chicago, IL, USA). For the Confirmatory factor analysis (CFA) and Exploratory Structural Equation Model (ESEM), R version 4.1.2 was used with the packages lavaan^42^ and GPArotation^43^. In all analyses, *p* values < .05 were set as thresholds for statistical significance. For the ICS, means were computed if there was a maximum of one missing item per subscale. Otherwise, respondents were excluded from the analyses of factorial validity, which was the case for five participants (0.01%). In all analyses, individual practitioner data were used. Mardia's multivariate skewness and kurtosis statistics^44^ was used to test multinormality, besides the Kaiser–Meyer–Olkin (KMO) test of sampling adequacy^45^ and Bartlett’s test of sphericity^46^. While both the latter indicated suitability of the data for factor analysis (KMO = 0.879, χ^2^ = 4443.55, *df* = 153,* p* < .001), the significant Mardia’s test statistics for skewness (*p* < .001) and kurtosis (*p* < .001) indicated multivariate normality deviation, leading to the use of robust estimation methods.

Standard item analyses were calculated, including item difficulties, corrected item-whole correlations, and Cronbach’s alpha if an item is deleted. The item difficulties—which in the context of measurement of attitudes can be interpreted as the mean endorsement of the respective item—was calculated as the actual endorsement of the item (sum of all participants’ scores) divided by the maximum possible endorsement (participant number * maximum score for any individual participant)^47^. This calculation results in an item difficulty p_i_ between 0 and 1, with 0 signifying no endorsement and 1 maximum endorsement. Values of p_i_ < 0.20 are regarded as low, values of 0.20 < p_i_ < .80 as medium and diagnostically ideal because they differentiate best between high and low endorsers and items of p_i_ > 0.80 as high, i.e. an item that generally was endorsed by most respondents^48^.

To obtain internal reliability coefficients of the scales and subscales, McDonald’s omega ω^49^ was calculated with 95% bias-corrected and accelerated bootstrap confidence intervals^50^ and 10 000 bootstrap replications using the R package MBESS^51^.

To assess construct validity, the factorial validity of the German version of ICS was examined by comparing the model fits of an independent cluster model of CFA (ICM-CFA), a Bifactor CFA, a Second-order CFA, an ESEM and a Bifactor ESEM. The maximum likelihood estimation method with robust standard error estimator was used with full-information maximum likelihood to handle missingness. The root mean square error of approximation (RMSEA), standardized root mean squared residual (SRMR), comparative fit index (CFI), and the ratio of χ^2^ to *df* were assessed as fit indices. RMSEA < 0.06 to 0.08 with confidence interval, SRMR ≤ 0.08, CFI ≥ 0.95 and χ^2^/*df* ≤ 2 or 3, indicate acceptable fit^52^. The Akaike Information Criterion (AIC) was computed to compare the models.

Thereafter, a multiple-group CFA was conducted with the Second-order model and a multiple-group SEM with the ESEM model to test the measurement invariance between men and women and between licensed psychotherapists and psychotherapists in training. The Second-order CFA model was chosen since it demonstrated comparable model fit indices while at the same time it can be regarded as the theoretically assumed factor structure. Different levels of measurement invariance were tested by defining a baseline model to assess configural invariance with similar loading patterns across groups, a metric invariance model with equated factor loadings, a scalar invariance model, constraining factor loadings and item intercepts to be equal across groups, and a latent means model, constraining item and error residuals to be equal across groups^53^. Configural invariance can be assumed in case of a good fit of the baseline model and groups sharing significant loadings, metric invariance if the metric invariance model is not substantially worse than the baseline model, and scalar invariance if the third model is not substantially worse than the baseline model. As model fit index, the chi-square test statistic was conducted besides the RMSEA, SRMR and CFI. Differences in fit indices between models are assumed to be substantially if the chi-square test statistic is significant and ∆CFI ≥ 0.01^54^. Scalar invariance is required to be able to infer group differences on actual differences in the latent variable rather than on the measurement.

To assess the convergent validity of the ICS, the following hypothesis was tested by calculating Pearson correlation coefficients assuming content overlap of the TPB construct subjective norms and the organizational implementation climate: The ICS total scale shows a high positive correlation to the ISP-D-SN. Smaller correlations are assumed to exist with the ISP-D-A, ISP-D-PBC and ISP-D-BI. According to Cohen’s classification^55^, *r* = 0.50 indicates high, *r* = 0.30 medium and *r* = 0.10 low correlation effect sizes. Group differences between men and women and between licensed psychotherapists and psychotherapists in training on the ICS were assessed with independent* t*-tests. In light of the unbalanced sample sizes^56,57^, Welch’s^58^ unequal variances *t*-test was used.

Pearson coefficients were calculated to assess correlations between ICS and EBPAS-36D as well as between age and the ICS. The latter result was compared to partial correlations between age and ICS total scale when controlling for license status, current occupation (in- or outpatient services) and EBPAS-36D total score. The findings are reported following the STrengthening the Reporting of Observational Studies in Epidemiology (STROBE) guideline^59^ and informed by the COnsensus-based Standards for the selection of health Measurement INstruments (COSMIN) taxonomy^60^.

### Ethical approval and consent to participate

The study was approved by the Internal Review Board of the University of Marburg (approval number: 2019-58 k). Participants received study information and provided informed consent. Data were collected anonymously.

## Results

### Item analysis

The item difficulties of the ICS ranged between *p*_i_ = 0.09 (item 12) and *p*_i_ = 0.69 (item 17) with a mean difficulty of *p*_i_ = 0.47. The mean inter-item correlation was *r* = 0.34. The items 10, 11 and 12 show markedly lower difficulties (*p*_i_ = 0.09 to *p*_i_ = 0.15) than all other items. The item-total correlations of the individual items with the total scale ranged from *r*_itc_ = 0.28 (item 12) to *r*_itc_ = 0.77 (item 15) with a mean item-total correlation of *r*_itc_ = 0.55. Only item 12 showed a *r*_itc_ < 0.30 (see Table 3). The correlations of the individual items with their subscales ranged from *r*_itc_ = 0.46 (item 18) to *r*_itc_ = 0.85 (item 3).

**Table 3:** Item analyses of ICS.

Item	Short description	*M* (*SD*)	*p*_i_	*r*_itc total_	α_total if deleted_	*r*_itc subscale_	α_subscale if deleted_
1	One of the goals is to use EBP	2.48 (1.05)	.62	.647	.90	.827	.88
2	Think implementation is important	2.63 (0.97)	.66	.665	.90	.814	.89
3	Using EBP is a top priority	2.36 (1.10)	.66	.696	.90	.849	.86
4	Provides conferences, workshops	2.08 (1.40)	.52	.597	.90	.785	.72
5	Provides EBP trainings or in-services	2.12 (1.34)	.53	.640	.90	.791	.71
6	Provides training materials, journals	2.15 (1.28)	.54	.553	.90	.581	.90
7	Clinicians are seen as clinical experts	2.33 (1.19)	.58	.673	.90	.723	.64
8	Clinicians are held in high esteem	2.35 (1.14)	.59	.665	.90	.707	.66
9	More likely to be promoted	1.21 (1.15)	.30	.567	.90	.510	.86
10	Provides financial incentives	0.60 (0.92)	.15	.415	.90	.643	.68
11	More likely to get a bonus or a raise	0.43 (0.77)	.11	.402	.90	.673	.65
12	Ability to accumulate compensated time	0.34 (0.76)	.09	.284	.91	.554	.77
13	Selects staff who previously used EBP	1.27 (1.25)	.32	.616	.90	.699	.79
14	Selects staff who have formal education	1.61 (1.32)	.40	.611	.90	.731	.75
15	Selects staff who value EBP	1.86 (1.27)	.47	.774	.89	.685	.80
16	Selects staff who are adaptable	2.67 (1.04)	.67	.388	.90	.687	.61
17	Selects staff who are flexible	2.74 (0.99)	.69	.319	.91	.701	.60
18	Selects staff open to new interventions	2.52 (1.04)	.63	.439	.90	.464	.85

Valid *n*: 421. *EBP* Evidence-based Practice; *ICS* Implementation Climate Scale; *p*_i_: Item Difficulty, *r*_itc_: Corrected item-whole correlation.

### Reliability

The internal consistency of the total scale was ω = 0.91 [0.89; 0.92]. Internal consistencies of the original model ICS subscales were: Focus on EBP ω = 0.92 [0.90; 0.93]; Educational Support for EBP ω = 0.86 [0.84; 0.89]; Recognition for EBP ω = 0.82 [0.78; 0.84]; Rewards for EBP ω = 0.79 [0.72; 0.84]; Selection for EBP ω = 0.84 [0.81; 0.87]; Selection for Openness ω = 0.80 [0.75; 0.84].

### Subscale correlations

The correlation coefficients between the ICS total scale and the six subscales are presented in Table 4. All subscale correlations were significant. The highest correlation was between the total scale and the Selection for EBP subscale (*r* = 0.83).

**Table 4:** Pearson correlations of ICS total scale, ICS subscales and age.

	1	2	3	4	5	6	7	8
1 ICS Total scale	–	.772**	.759**	.816**	.510**	.828**	.543**	− .113*
2 ICS Focus on EBP	422	–	.622**	.602**	.211**	.522**	.254**	− .139**
3 ICS Educational support for EBP	422	425	–	.511**	.238**	.474**	.247**	− .131**
4 ICS Recognition for EBP	422	423	423	–	.388**	.637**	.319**	− .073**
5 ICS Rewards for EBP	422	422	422	422	–	.422**	.126**	.022**
6 ICS Selection for EBP	422	424	424	423	422	–	.438**	− .078**
7 ICS Selection for openness	422	425	425	423	422	424	–	− .051**
8 Age	422	425	425	423	422	424	425**	–

*ICS* Implementation Climate Scale. Upper half: Pearson correlation coefficient, *p < .05 **p < .001; Lower half: Sample sizes.

### Validity

#### Model comparison

The path diagrams of the models (ICM-CFA, Bifactor CFA, Second-order CFA, ESEM and Bifactor ESEM) including their standardized regression coefficients are shown in the Supplemental material 2. All model fit indices are found in Table 5. It should be noted that for the ESEM and the Bifactor ESEM, the variance–covariance matrices of the estimated parameters were not positive definite probably indicating non-identified models.

**Table 5:** Comparison of model fit indices.

Model	χ^2^	*df*	*p*	χ^2^/*df*	RMSEA [90% CI]	SRMR	CFI	AIC
ICM-CFA	370.77	120	< .001	3.09	.070 [.062; .079]	.067	.94	18,811.15
Bifactor CFA	308.94	117	< .001	2.64	.062 [.054; .071]	.057	.96	18,755.31
Second-order CFA	449.79	129	< .001	3.49	.077 [.069; .085]	.078	.93	18,872.17
ESEM	93.04	36	.006	2.58	.061 [.046; .077]	.012	.99	18,701.42
Bifactor ESEM	56.31	34	.009	1.66	.039 [.020; .057]	.010	.99	18,668.69

*N* = 421. *CFA* Confirmatory factor analysis. *ESEM* Exploratory structural equation modeling. *RMSEA* Root mean square error of approximation, *SRMR* Standardized root mean residual, *CFI* Comparative fit index, *AIC* Akaike information criterion.

In the ICM-CFA and the Second-order CFA, all regression weights were significant. In the Bifactor CFA, the regression weights of item 9 on the Recognition for EBP subscale was non-significant. In the ESEM, significant regression weights were found for the items 1, 2 and 3 on the Focus on EBP subscale, for the items 4, 5 and 6 on the Educational Support for EBP subscale, for the items 7 and 8 on the Recognition for EBP subscale, for item 11 on the Rewards for EBP subscale and for items 16, 17 and 18 on the Selection for Openness subscale. In the Bifactor ESEM, significant regression weights were found for the items 1, 2 and 3 on the Focus on EBP subscale, for the items 4, 5 and 6 on the Educational Support for EBP subscale, for item 10 on the Rewards for EBP subscale, for items 13 and 14 on the Selection for EBP subscale and for the item 17 on the Selection for Openness subscale. All items except the items 9, 10, 11, 12, 16, 17 and 18 show significant regression weights on the Implementation Climate scale.

The model fit indices (see Table 5) indicated adequate fit for the ICM-CFA (RMSEA [90% CI] = 0.070 [0.062; 0.079], SRMR = 0.067, CFI = 0.94), the Bifactor CFA (RMSEA [90% CI] = 0.062 [0.054; 0.071], SRMR = 0.057, CFI = 0.96), the Second-order CFA (RMSEA [90% CI] = 0.077 [0.069; 0.085], SRMR = 0.078, CFI = 0.93) and slightly better model fits for the ESEM (RMSEA [90% CI] = 0.061 [0.046; 0.077], SRMR = 0.012, CFI = 0.99) and the Bifactor ESEM (RMSEA [90% CI] = 0.039 [0.020; 0.057], SRMR = 0.010, CFI = 0.99). However, the latter models demonstrated less parsimony with significantly fewer degrees of freedom (*df* = 34–36) compared to the other models (*df* = 117–129). The AICs of the models were not substantially different, ranging from 18,668.69 (Bifactor ESEM) to 18,872.17 (Second-order CFA).

#### Measurement invariance

A multiple-group CFA based on the Second-order CFA model and a multiple-group SEM based on the ESEM model were used to test for measurement invariance in the ICS across males and females as well as across licensed psychotherapists and psychotherapists in training. The results of the CFA and ESEM approach do not differ substantially. Fit indices and chi-square test statistics for all measurement models are found in Table 6. Adequate model fits for all measurement models were found. Non-significant χ^2^ difference test statistics and ∆CFI < 0.01 indicate no substantial differences between the configural, metric and scalar models for both gender and psychotherapy license comparisons, allowing to assume measurement invariance. Differences emerge across licensed psychotherapists and psychotherapists in training comparing the scalar model and the residual invariance model, indicating latent means invariance is not given.

**Table 6:** Multiple-group Second-order CFA and ESEM model fit indices.

Model	χ^2^ (∆χ^2^)	*df* (∆*df*)	*p* (*∆p*)	RMSEA (∆RMSEA)	CFI (∆CFI)
Gender					
Second-order CFA					
0 Configural	613.01	258	< .001	.081	.919
1 Metric	624.95 (11.94)	275 (17)	< .001 (.804)	.078 (.003)	.920 (.001)
2 Scalar	632.84 (7.89)	286 (11)	< .001 (.723)	.076 (.002)	.921 (.001)
3 Latent means	640.13 (7.29)	293 (7)	< .001 (.399)	.075 (.001)	.921 (< .001)
ESEM					
0 Configural	165.98	72	< .001	.079	.979
1 Metric	255.82 (89.84)	174 (102)	< .001 (.799)	.047 (.032)	.981 (.003)
2 Scalar	267.52 (11.70)	186 (12)	< .001 (.470)	.046 (.002)	.981 (< .001)
3 Latent means	274.69 (7.17)	192 (6)	< .001 (.305)	.045 (< .001)	.981 (< .001)
Psychotherapy license					
Second-order CFA					
0 Configural	610.97	258	< .001	.081	.920
1 Metric	637.35 (26.37)	275 (17)	< .001 (.068)	.079 (.002)	.917 (.002)
2 Scalar	651.14 (13.79)	286 (11)	< .001 (.245)	.078 (.001)	.917 (.001)
3 Latent means	669.17 (18.03)	293 (7)	** < .001 (.012)**	.078 (< .001)	.914 (.003)
ESEM					
0 Configural	174.05	72	< .001	.082	.977
1 Metric	267.13 (93.07)	174 (102)	< .001 (.725)	.050 (.032)	.979 (.002)
2 Scalar	280.07 (12.94)	186 (12)	< .001 (.373)	.049 (.001)	.979 (< .001)
3 Latent means	295.75 (15.69)	192 (6)	** < .001 (.016)**	.051 (.002)	.976 (.002)

*N* = 420. Gender: *n* = 359 female, *n* = 61 male. Psychotherapy license: *n* = 333 without license,* n* = 87 with license. *CFA* Confirmatory factor analysis. *ESEM* Exploratory structural equation modeling. *RMSEA* Root mean square error of approximation, *CFI* Comparative fit index.

#### Correlation analyses

Table 7 shows the results of the correlation analyses between the ICS total scale and the ISP-D scales. As expected, a high positive correlation between the ICS total scale and the ISP-D-SN was found (*r* = 0.590**, *p* < 0.001, *n* = 403). Correlations to all other ISP-D scales were significant, yet smaller (see Table 7).

**Table 7:** Pearson correlations of ICS total scale and the ISP-D scales.

	1	2	3	4	5
1 ICS Total scale	−	− .360**	.590**	− .212**	.442**
2 ISP− D Attitudes	408	−	− .507**	.048	.675**
3 ISP− D Subjective norms	403	406	−	− .294**	.682**
4 ISP− D Perceived behavioral control	396	398	398	−	− .093
5 ISP− D Behavioral intention	393	395	394	392	−

*Notes.* ICS: Implementation Climate Scale. *ISP-D* Intention Scale for Providers Direct Items. Upper half: Pearson correlation coefficients, * *p* < .05 ** *p* < .001; Lower half: Sample sizes.

### Group differences and correlations

No significant group differences were found between men and women on the ICS total scale or any subscale. Age showed a slight (but significant) negative correlation with the ICS total scale (*r* = -0.11) and with the subscales Focus on EBP (*r* = -0.14) and Educational support for EBP (*r* = -0.12) (see Table 4). This indicates more favorable ICS ratings on these subscales for younger participants.

Participants in postgraduate training more frequently reported working in outpatient services (*n* = 189) than in inpatient services (*n* = 141), while licensed psychotherapists’ current occupation was more balanced (*n* = 26 resp. *n* = 34; χ^2^ = 3.99, *df* = 1, *p* = 0.046). Since psychotherapists in training are typically younger than licensed psychotherapists (*t* = -8.12, *df* = 106.93, *p* < 0.001), participants working in outpatient services were younger than those working in inpatient services (*t* = -2.12, *df* = 358.25, *p* = 0.035). Therefore, license status and/or current occupation might explain the association between ICS ratings and age. However, the correlation between age and ICS total scale was still significant when controlling for license status (*r* = -0.13, *p* = 0.009) and current occupation in in- or outpatient services (*r* = -0.15, *p* = 0.002). However, group differences were found for the ICS subscale Educational Support for EBP between psychotherapists in training (*M* = 3.19, *SD* = 1.16) and licensed psychotherapists (*M* = 2.89, *SD* = 1.19, *t* = 2.05, *df* = 131.02, *p* = 0.032, *d* = 0.25). Moreover, licensed psychotherapists and psychotherapists in training differed in their ratings for some ICS subscales on inpatient and outpatient services (see Table 8 and 9).

**Table 8:** Group differences on ICS between in- or outpatient services rated by licensed psychotherapists.

	Inpatient (*n* = 34)		Outpatient (*n* = 26)					
	*M*	*SD*	*M*	*SD*	*t*	*df*	*p*	*d*
Total score	2.83	0.65	2.63	0.85	0.99	45.62	.330	0.26
Focus on EBP	3.26	1.00	3.17	0.86	0.41	57.20	.686	0.10
Educational support for EBP	3.05	0.99	2.33	1.27	2.38	46.40	.022*	0.63
Recognition for EBP	2.98	1.08	2.85	1.21	0.45	50.46	.657	0.11
Rewards for EBP	1.47	0.68	1.53	0.68	− 0.31	53.80	.757	− 0.09
Selection for EBP	2.44	0.96	2.54	1.20	− 0.34	47.14	.736	− 0.09
Selection for openness	*3.78*	*0.68*	*3.40*	*0.93*	*1.79*	*43.75*	*.081*	*0.47*

*EBP* Evidence-based Practice; *ICS* Implementation Climate Scale. * *p* < .05 ** *p* < .001.

**Table 9:** Group differences on ICS between in- or outpatient services rated by psychotherapists in training.

	Inpatient (*n* = 141)		Outpatient (*n* = 189)					
	*M*	*SD*	*M*	*SD*	*t*	*df*	*p*	*d*
Total score	2.82	0.64	2.91	0.69	− 1.12	310.30	.265	− 0.14
Focus on EBP	3.34	0.97	3.65	0.92	− 2.90	291.22	.004*	− 0.33
Educational support for EBP	3.00	1.03	3.32	1.23	− 2.56	324.00	.011*	− 0.28
Recognition for EBP	2.88	0.91	2.98	0.94	− 0.98	306.56	.326	− 0.11
Rewards for EBP	1.54	0.70	1.35	0.64	2.57	282.34	.011*	0.28
Selection for EBP	2.62	1.00	2.49	1.14	1.05	319.18	.295	0.12
Selection for openness	3.61	0.78	3.64	0.93	− 0.35	323.50	.730	− 0.03

*EBP* Evidence-based Practice; *ICS* Implementation Climate Scale. *p < .05 **p < .001.

The ICS total scale correlated positively with the EBPAS-36D total scale (*r* = 0.41, *p* < .001, *n* = 404). When controlling for participants’ scores on the EBPAS-36D total scale, the correlation between age and ICS total scale was no longer significant (*r* = -− .02, *p* = .639).

## Discussion

Based on survey data from a sample of German psychotherapists, our results demonstrated good item properties and internal consistencies as well as factorial and convergent validity for the German version of the ICS.

Item analyses indicate that most item difficulties were in the medium range. This is desirable as it allows optimal differentiating between respondents. Still, three items of the subscale Rewards for EBP on the ICS (items 10, 11, 12) received less endorsement than the other items. The items 10 and 11 assess the provision of financial incentives (bonuses or pay rises) for the use of EBP, item 12 assesses the ability to accumulate compensated time for the use of EBP. In the German mental health care system, financial incentives, bonuses and pay rises or the possibility to accumulate compensated time are rarely employed implementation strategies. Among samples in the USA^14,25,26^ and Norway^30^, scores on the Rewards subscale have been very low as well. Engell et al.^29^ also attributed this to the fact that there are no systematic practices for financial rewards for EBP use in Norwegian child welfare services. Removing item 12 (Ability to accumulate compensated time) and 17 (Selects staff who are flexible) would even result in a slight improvement (0.01) of the internal consistency of the total scale. However, removing these items might affect the subscales’ content validity and the comparability with the original scale so the items were retained in this version. Moreover, these items allow a comparison with other health care systems where such use of incentives is already practiced.

Despite these minor item issues, the internal consistency of the ICS total scale is excellent (ω = 0.91) and comparable to those in previous studies (α = 0.87 to α = 0.89)^28,29^. The subscale’s internal consistencies ranged from acceptable to excellent, with the Rewards for EBP and the Selection for Openness subscales demonstrating the lowest internal consistencies. Future studies should investigate whether respondents interpret the items of the Rewards for EBP subscale in the same ways in order to rule out misunderstandings. This could be accomplished through cognitive interviewing in order to assess how clinicians perceive and interpret specific items and constructs^61,62^.

The results indicate factorial validity with adequate model fits for an ICM-CFA, Bifactor CFA and Second-order CFA and even better model fits for an ESEM and a Bifactor ESEM. However, both ESEM models demonstrated less sparsity, non-positive definite variance–covariance matrices and non-significant regression weights. Accordingly, we prefer the Second-order model with six factors that can be regarded as the theoretically assumed model. It demonstrated significant regression weights and an adequate model fit (RMSEA [90% CI] = 0.077 [0.069; 0.085], SRMR = 0.078, CFI = 0.93). Convergent validity of the ICS is supported by confirming the expected high positive correlations with two scales assessing the TPB construct of subjective norms to use EBP. Convergent validity as one type of construct validity supports the assumption of cross-cultural validity of the ICS, insofar that the ICS can be used to assess the Implementation Climate Scale in the German mental health care system despite differences in the health systems and frameworks for the provision of mental health care.

The results of a multiple-group CFA and a multiple-group ESEM allow the assumption of measurement invariance of the ICS across gender groups and across psychotherapists in training and licensed psychotherapists. Only residual invariance (latent means) is not given across licensed psychotherapists and psychotherapists in training. However, this is inconsequential to calculating and interpreting group mean differences when scalar invariance is found^63^. Therefore, we were able to assess potential group differences. No gender differences were found for the ICS, but respondents’ age showed small negative correlations with the ICS total scale, Focus on EBP and Educational support for EBP subscale—suggesting that the younger a psychotherapist was, the more favorable he or she judged the implementation climate. One potential explanation for the identified age differences might be the type of organization younger psychotherapists predominantly work in. We found that the younger group of psychotherapists in training more frequently work in outpatient services than older and licensed psychotherapists. Moreover, their ratings on inpatient and outpatient clinics’ implementation climate diverges from those of licensed psychotherapists. Besides the type of organization making a difference, psychotherapists in training might differ in their perceptions of the organizations’ implementation climate—or might be treated differently from their licensed colleagues. Future studies should examine these differences in more detail. Still, the correlation between participants’ age and the ICS total scale was significant even after controlling for their license and current occupation, but was no longer significant when controlling for their attitudes towards EBP. In line with previous studies^64,65^, the organizations’ implementation climate was associated with psychotherapists’ attitudes towards EBP in our study. The higher they rated the implementation climate of their organization or work group, the more positive was their attitude towards EBP. Younger psychotherapists report more positive attitudes towards EBP^39^ and consider EBP implementation as more important than older therapists. Therefore, the younger psychotherapists are, the more they may be aware of efforts undertaken on the side of the organization or work group to foster EBP implementation.

Some limitations of our study should be considered when interpreting the results. One major limitation is that due to the open nature of our online study and in order prevent concerns of insufficient anonymity, we did not ask participants to disclose the name of their organization or work group. Accordingly, we could not allocate ratings to one or another organization and assess interrater agreement within organizations. Given sufficient within-group interrater agreement, the aggregation of individual-level data to organizational level data would enable to assess the implementation climate construct as the shared characteristic within organizations. Accordingly, future studies should build on our results demonstrating good psychometric properties of the ICS at the individual level to examine its suitability for investigation at the organizational level. Yet, individual perceptions of the implementation climate might well predict EBP use, especially if implementation climate on the organizational level is weak, e.g. there is high within-group variability in the perceptions of the climate^66^.

Another limitation of our study is that even through advertised widely and via obligatory professional associations, the online sample constitutes a convenience sample. A large proportion of the sample reported being in training to become psychotherapists. Participants predominantly subscribed to a CBT approach and are not representative of the population of mental health providers in Germany: Nübling et al.^67^ report that among licensed psychotherapists offering mental health care in private practices with reimbursement by the statutory health insurance, therapy approaches are 50.6% PDT, 49.0% CBT, and 25.6% psychoanalysis. In light of the dropout rate in the present study, although in the average range for online surveys^68^, participants may have been more likely to complete the survey if they were particularly interested in its topic^69^. Although this does not affect the psychometric investigation of the ICS, future studies should aim to recruit more representative samples.

## Conclusions

Organizations’ implementation climate is an important construct in implementation research and implementation efforts benefit from its consideration. The ICS is a pragmatic and psychometrically strong measure assessing employees’ perceived importance of EBP implementation within the organization or work group. Although further validating research is required, the present study confirms good psychometric properties and validity of a German version of the ICS in an online recruited convenience sample of psychotherapists.

## Supplementary Information

Supplementary Information 1.

Supplementary Information 2.

Supplementary Information 3.

## Data Availability

All data generated or analysed during this study—without potentially identifying socio-demographic and occupational information—are included in this published article and its supplementary information files (Supplemental material 3).

## Abbreviations

AIC: Akaike information criterion
CBT: Cognitive behavioral therapy
CFA: Confirmatory factor analysis
CFI: Comparative fit index
COSMIN: COnsensus-based Standards for the selection of health Measurement Instruments
EBP: Evidence-based practices
EBPAS: Evidence-based practice attitudes scale
EPIS: Exploration, preparation, implementation, sustainment framework
ESEM: Exploratory structural equation model
ICM-CFA: Independent cluster model of confirmatory factorial analysis
ICS: Implementation Climate Scale
ISP: Intention scale for providers
RMSEA: Root mean square error of approximation
SRMR: Standardized root mean squared residual
STROBE: STrengthening the reporting of observational studies in epidemiology
TPB: Theory of planned behavior
