# Supplementary material for: Climate for evidence-based mental health care implementation in Germany: psychometric investigation of the Implementation Climate Scale (ICS)
Source: Sci Rep. 2023 Mar 31;13:5311. doi: 10.1038/s41598-023-32282-4 (PMC10066389; doi:10.1038/s41598-023-32282-4)
Supplement: Supplementary file 1 — Supplementary Information 1. [file 41598_2023_32282_MOESM1_ESM.pdf]

Supplemental material

Supplemental material 1

German ICS Translation and Scoring Instruction (Supplemental material 1.docx)

Skala zur Erfassung des Implementationsklimas (ICS)

Deutschsprachige Version der Implementation Climate Scale (Ehrhart et al., 2014)

Evidenzbasierte Methoden sind Behandlungs- oder Interventionsmethoden (in der Psychotherapie z.B. bestimmte Therapiemanuale, in der Körpermedizin z.B. Medikamente oder Operationsverfahren), deren Wirksamkeit empirisch in verschiedenen wissenschaftlichen Studien nachgewiesen wurde. Dies kann z.B. geschehen, indem die Wirksamkeit einer Psychotherapie gegenüber der einer Wartelistenbedingungen oder einer alternativen Behandlung nachgewiesen wurde.

Bitte geben Sie an, in welchem Ausmaß Sie jeder Aussage zustimmen und nutzen Sie das untenstehende Antwortformat.

**0**	**1**	**2**	**3**	**4**
**Überhaupt keine Zustimmung**	**Geringe Zustimmung**	**Moderate Zustimmung**	**Starke Zustimmung**	**Sehr starke Zustimmung**

1. Eines der Hauptziele der Einrichtung ist der effektive Einsatz evidenzbasierter Methoden.....0 1 2 3 4

2. Mitarbeiterinnen und Mitarbeiter der Einrichtung denken, dass die Implementierung evidenzbasierter Methoden wichtig ist...0 1 2 3 4

3. Der Einsatz evidenzbasierter Methoden hat eine sehr hohe Priorität in dieser Einrichtung......0 1 2 3 4

4. Die Einrichtung bietet Konferenzen, Workshops oder Seminare zu evidenzbasierten Methoden an...0 1 2 3 4

5. Die Einrichtung bietet Schulungen oder Weiterbildungen zu evidenzbasierten Methoden an...0 1 2 3 4

6. Die Einrichtung bietet Schulungsmaterialien, Zeitschriften etc. zu evidenzbasierten Methoden an...0 1 2 3 4

7. Fachkräfte in der Einrichtung, die evidenzbasierte Methoden einsetzen, werden als (klinische) Expertinnen und Experten angesehen...0 1 2 3 4

8. Fachkräfte, die evidenzbasierte Methoden einsetzen, werden in dem Team/ der Einrichtung sehr wertgeschätzt...0 1 2 3 4

9. Fachkräfte in der Einrichtung, die evidenzbasierte Methoden einsetzen, werden eher befördert..0 1 2 3 4

10. Die Einrichtung bietet finanzielle Anreize für den Einsatz evidenzbasierter Methoden.........0 1 2 3 4

11. Je mehr evidenzbasierte Methoden man einsetzt, desto eher erhält man einen Bonus oder eine Beförderung...0 1 2 3 4

12. Die Einrichtung bietet die Möglichkeit, bezahlte Überstunden für den Einsatz evidenzbasierter Methoden anzusammeln..0 1 2 3 4

13. Die Einrichtung wählt Mitarbeiterinnen und Mitarbeiter aus, die schon evidenzbasierte Methoden eingesetzt haben..0 1 2 3 4

14. Die Einrichtung wählt Mitarbeiterinnen und Mitarbeiter aus, die eine offizielle Fortbildung absolviert haben, die evidenzbasierte Methoden unterstützt..0 1 2 3 4

15. Die Einrichtung wählt Mitarbeiterinnen und Mitarbeiter aus, die evidenzbasierte Methoden wertschätzen..0 1 2 3 4

16. Die Einrichtung wählt Mitarbeiterinnen und Mitarbeiter aus, die anpassungsfähig sind........0 1 2 3 4

17. Die Einrichtung wählt Mitarbeiterinnen und Mitarbeiter aus, die flexibel sind.....................0 1 2 3 4

18. Die Einrichtung wählt Mitarbeiterinnen und Mitarbeiter aus, die offen für neue Behandlungs- oder Interventionsformen sind..0 1 2 3 4

Auswertungshinweise

Der Score für jede Subskala wird erstellt, indem der Mittelwert der jeweiligen Items einer Subskala berechnet wird. Der Mittelwert der Zustimmung zu Item 1, Item 2 und Item 3 ergibt die Subskala 1 (Fokus). Die Gesamtscore wird erstellt, indem der Mittelwert der Scores aller Subskalen berechnet wird.

**Skala**	**Item Score**	**Score der Skala**
**Subskala 1: Fokus**		
Item 1		(Item1+Item2+Item3)/3
Item 2		
Item 3		
**Subskala 2: Unterstützung bei der Fortbildung**		
Item 4		(Item4+Item5+Item6)/3
Item 5		
Item 6		
**Subskala 3: Anerkennung**		
Item 7		(Item7+Item8+Item9)/3
Item 8		
Item 9		
**Subskala 4: Belohnungen**		
Item 10		(Item10+Item11+Item12)/3
Item 11		
Item 12		
**Subskala 5: Auswahl für EBP**		
Item 13		(Item13+Item14+Item15)/3
Item 14		
Item 15		
**Subskala 6: Auswahl für Offenheit**		
Item 16		(Item16+Item17+Item18)/3
Item 17		
Item 18		
**Gesamtskala**		(Skala1+Skala2+Skala3+ Skala4+Skala5+Skala6)/6
