# Supplementary figures and images for: Climate for evidence-based mental health care implementation in Germany: psychometric investigation of the Implementation Climate Scale (ICS)
Source: Sci Rep. 2023 Mar 31;13:5311. doi: 10.1038/s41598-023-32282-4 (PMC10066389; doi:10.1038/s41598-023-32282-4)

ICM-CFA

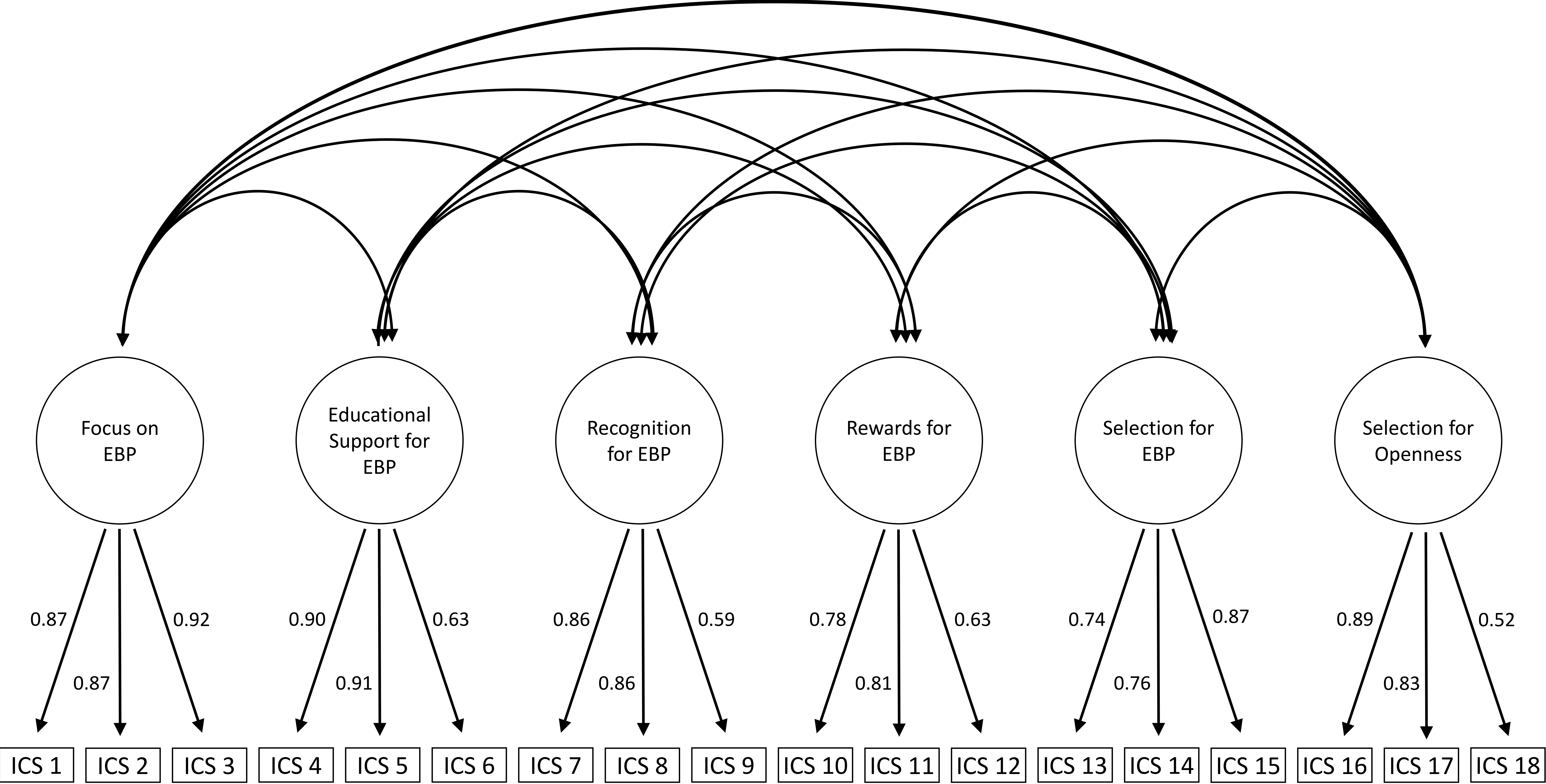

# Bifactor CFA

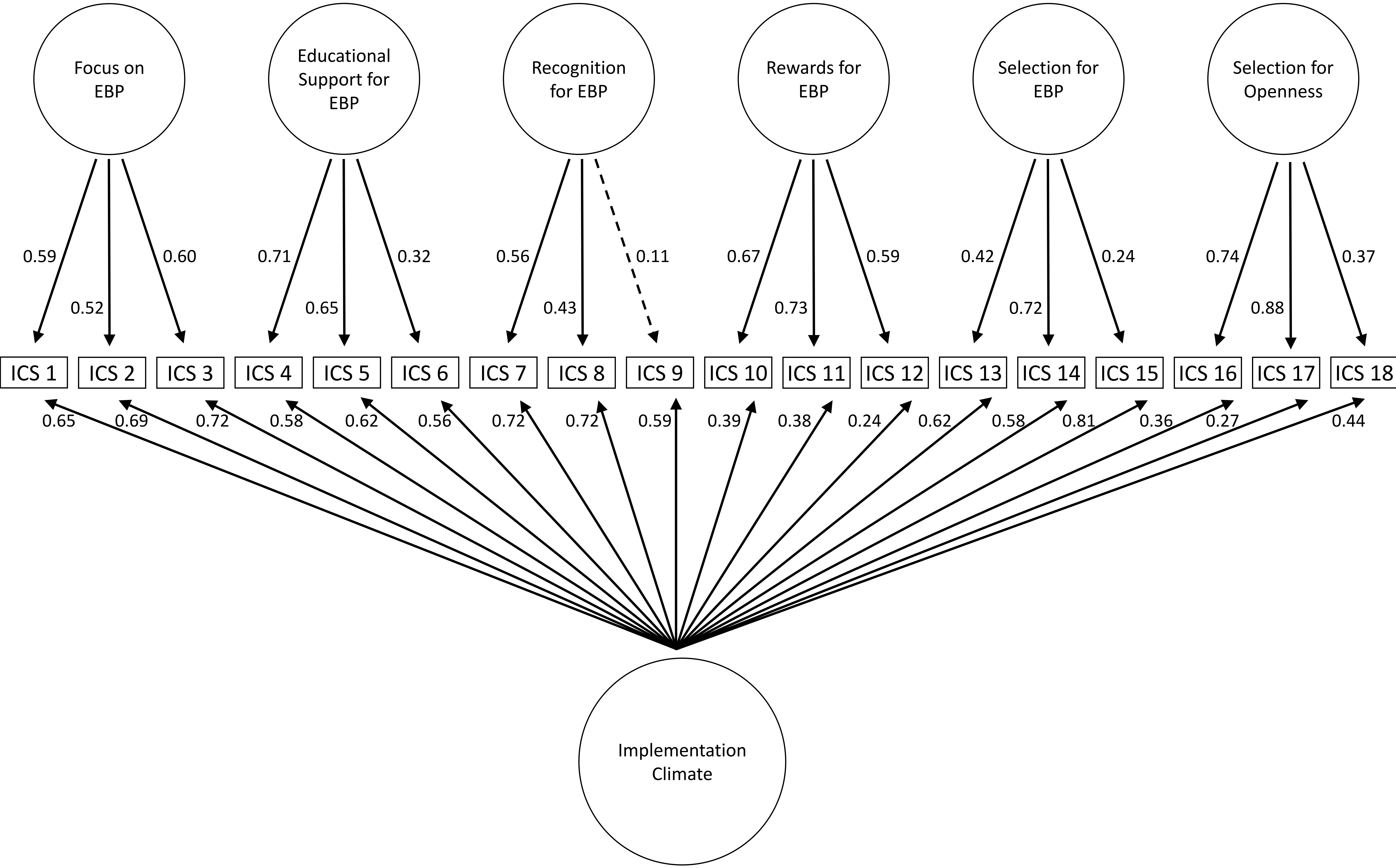

# Higher-order CFA

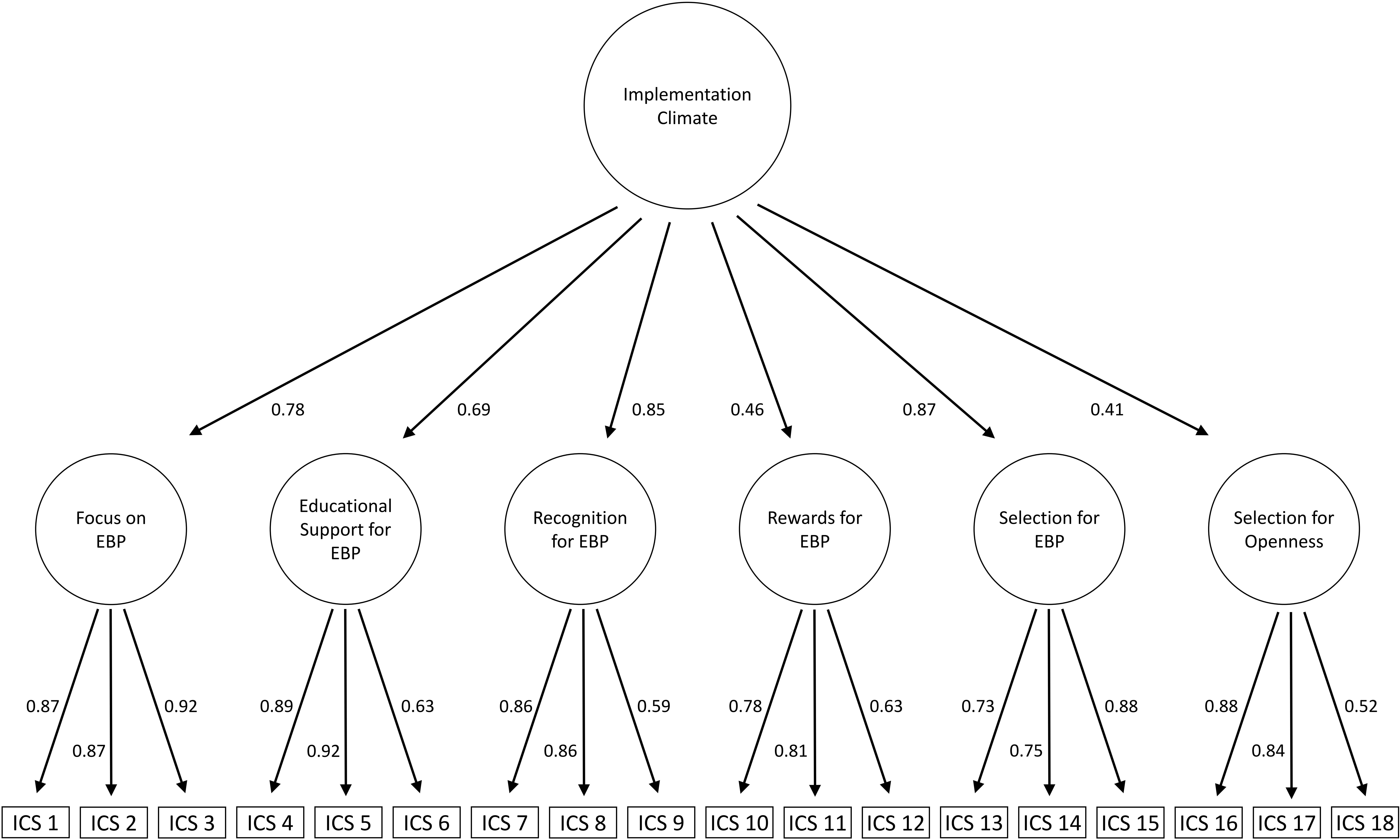

# ESEM

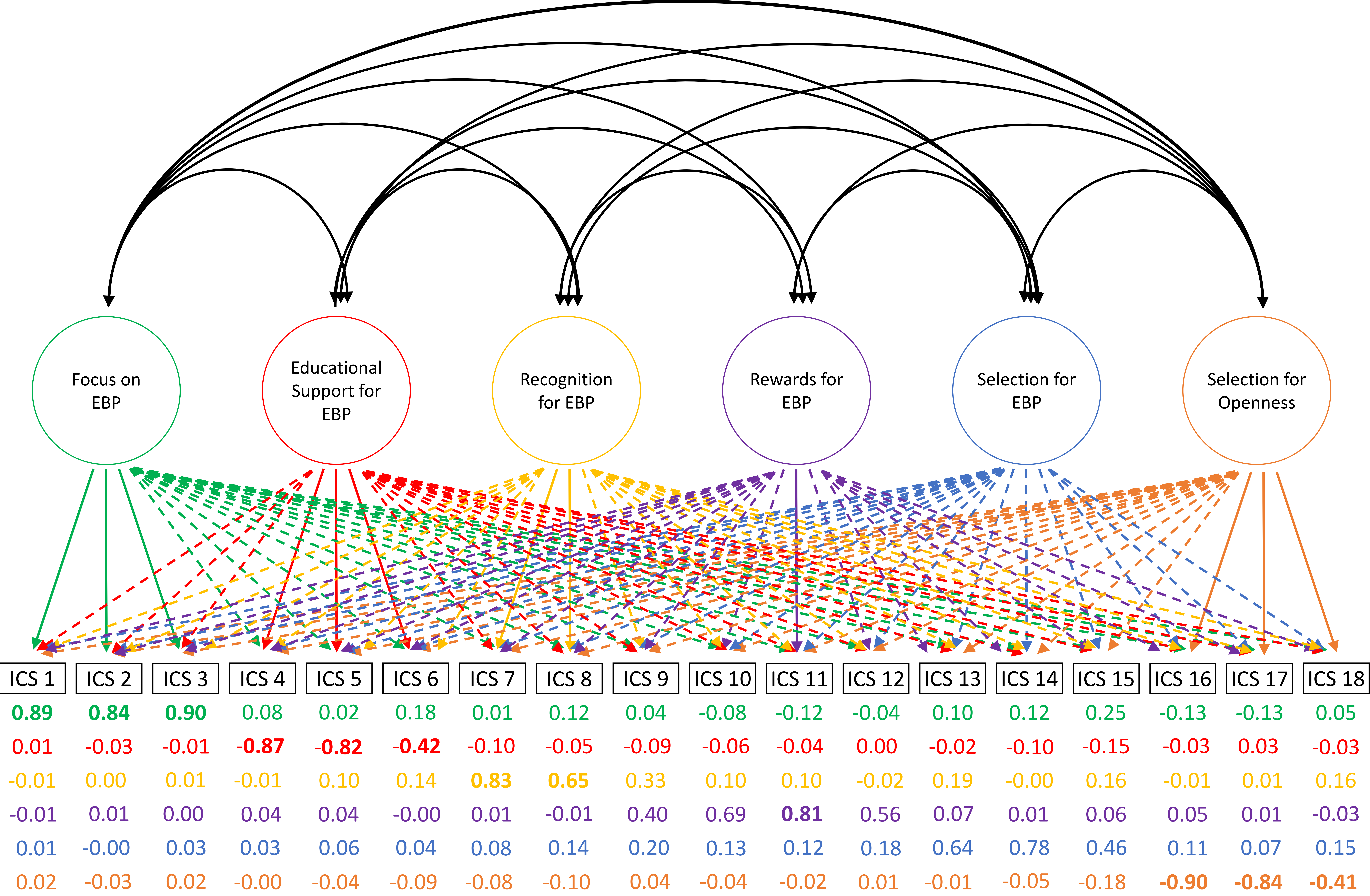

# Bifactor ESEM

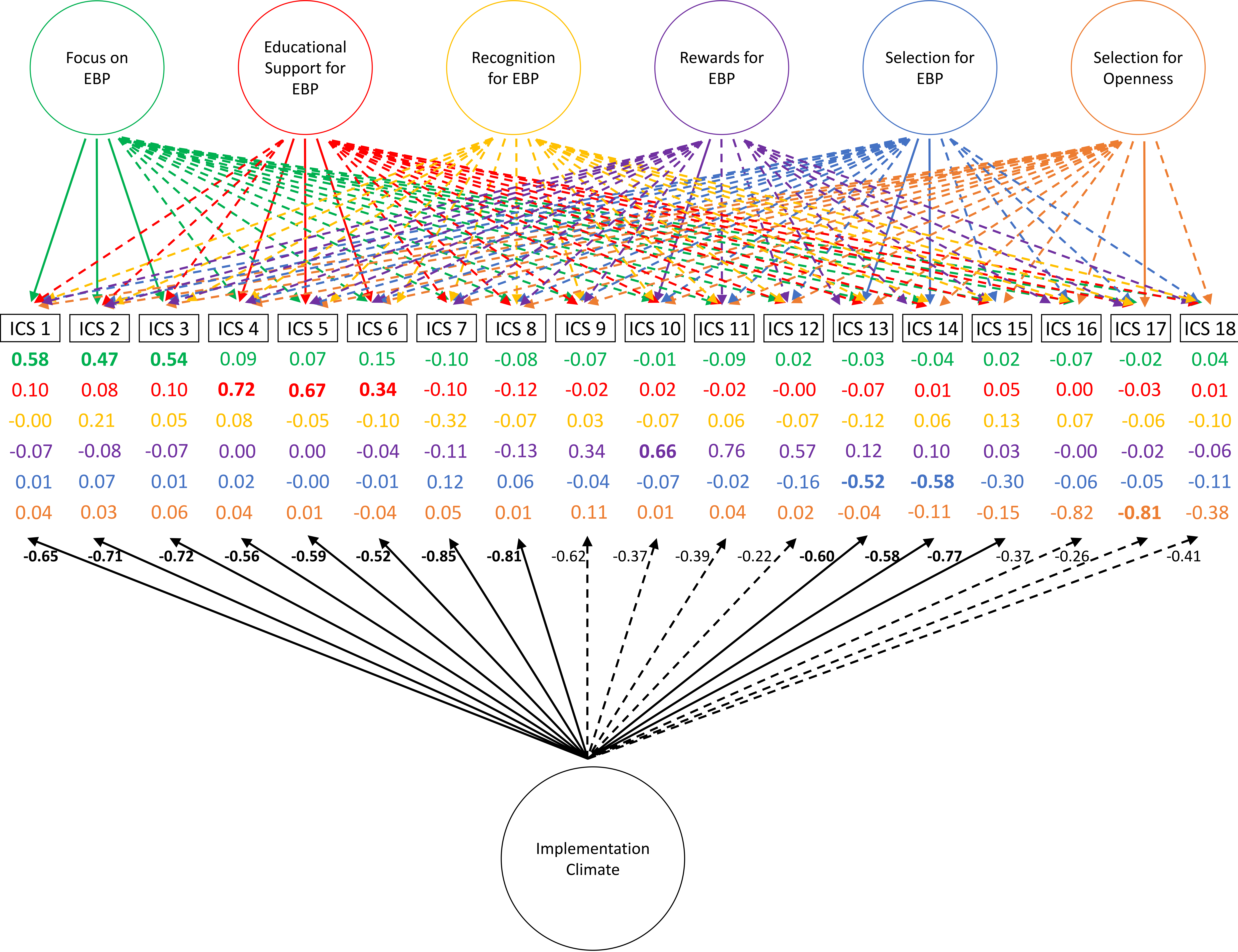

Supplement: Supplementary file 2 — Supplementary Information 2. [file 41598_2023_32282_MOESM2_ESM.pdf]
